# Performance of the Experimental EuroQol Toddler and Infant Populations (EQ-TIPS) and PedsQL in Infants and Toddlers with a Health Condition

**DOI:** 10.36469/001c.145813

**Published:** 2025-11-17

**Authors:** Janine Verstraete, Marco Zampoli, Alan Davidson, Marc Hendricks, Helder de Quintal, Yasmin Goga, Jo M. Wilmshurst, Alvin Ndondo, Gillian Riordan, Ronalda De Lacy, Mignon McCullogh, Deveshni Reddy, Lasse Herdien

**Affiliations:** 1 Paediatrics and Child Health Faculty of Health Sciences, University of Cape Town, South Africa; 2 Division of Pulmonology, Department of Paediatrics and Child Health Faculty of Health Sciences, University of Cape Town, South Africa; 3 Division of Pulmonology Red Cross War Memorial Children’s Hospital, Cape Town, South Africa; 4 Division of Haematology and Oncology Red Cross War Memorial Children’s Hospital, Cape Town, South Africa; 5 Division of Haematology and Oncology, Department of Paediatrics and Child Health Faculty of Health Sciences, University of Cape Town, South Africa; 6 University of CaRed Cross War Memorial Children’s Hospital, Cape Town, South Africa; 7 Red Cross War Memorial Children’s Hospital, Cape Town, South Africa; 8 Division of Haematology and Oncology, Department of Paediatrics and Child Health, Faculty of Health Sciences University of Cape Town, South Africa; 9 Red Cross War Memorial Children’s Hospital, Cape Town, South Africa; 10 Department of Paediatric Neurology Red Cross War Memorial Children’s Hospital, Cape Town, South A; 11 Neuroscience Institute, University of Cape Town, South Africa; 12 Department of Paediatric Neurology Red Cross War Memorial Children’s Hospital, Cape Town, South Africa; 13 Division of Gastroenterology, Department of Paediatrics and Child Health Faculty of Health Sciences, University of Cape Town, South Africa; 14 Division of Nephrology, Department of Paediatrics and Child Health University of Cape Town, South Africa; 15 Red Cross War Memorial Children’s Hospital; 16 Division of Nephrology, Department of Paediatrics and Child Health Faculty of Health Sciences, University of Cape Town, South Africa; 17 Department of Paediatrics and Child Health Faculty of Health Sciences, University of Cape Town, South Africa

**Keywords:** EQ-TIPS, PedsQL, infants, toddlers, health-related quality of life

## Abstract

**Background:**

Health-related quality of life measurement in infants and toddlers is increasingly important, but generic preference-weighted instruments lack evidence. This study compared the experimental EuroQol Toddler and Infant Populations (EQ-TIPS) and PedsQL in children 0 to 4 years.

**Methods:**

EQ-TIPS-3L v2.0 and PedsQL response distributions were compared by frequency. Item and dimension/summary score associations were computed using Pearson and intra-class correlation coefficient. Age and severity groups (EQ VAS ≥80) were compared with Mann-Whitney U tests.

**Results:**

Cross-sectional data from 260 children were analyzed: 0 to 24 months (n = 111) and 2 to 4 years (n = 149). Most caregivers were mothers, spending significantly more time (≥10 hours) with younger children χ2 = 18.12, *P* = .001). The EQ-TIPS-3L had the highest problems with eating (27%-31%) and pain (23%-25%) across age groups, with minimal missing data (≤1%). Younger children most frequently had problems with PedsQL: “tired” (54%), “resting a lot” (52%), “crying or fussing when left alone” (61%) and “difficulty soothing when upset” (51%). Older children’s main problems were “hurts or aches” (54%), “afraid or scared” (53%), “sad or blue” (50%), “angry” (64%) and “missing school” (56%-65%). All 3 of the PedsQL school items had missing data for older children (27%-30%). Hypothesized item correlations were reached for 30 of 35 and 11 of 12 items in the younger and older groups, respectively. EQ-TIPS-3L LSS showed moderate to strong correlations with all PedsQL scores except for cognitive (0-24 months) and school functioning (2-4 years). Both measures significantly differentiated by severity groups (EQ VAS ≥80) but not by age group.

**Conclusion:**

Both measures showed similar response distributions despite different time frames and response scales. EQ-TIPS-3L eating and pain reported high problems, with eating strongly associated only with PedsQL physical symptoms. The 2- to 4-year PedsQL version had many missing school functioning items; the 13- to 24-month PedsQL may suit older 2- to 4-year-olds better. Low association between PedsQL cognitive functioning and EQ-TIPS-3L suggests further research is needed on this potentially missing construct.

## BACKGROUND

There is a growing interest in the assessment of health-related quality of life (HRQoL) in infants and toddlers.^1^ It appears that this interest is in direct response to an increased focus on health interventions and technologies aimed at these young children^2^ and a corresponding call for age-appropriate and valid outcome measures.^3–5^

The Pediatric Quality of Life Inventory (PedsQL) 4.0 Generic Core Scales (GCS) were developed as generic non-preference-based measures to measure HRQoL in children.^6,7^ The scales measure HRQoL across 5 dimensions, including physical functioning (and symptoms in infants aged 1-24 months), emotional functioning, social functioning, and cognitive/school functioning. Each version has a variable number of items with the questionnaire for age 1 to 12 months, including 36 items; 13 to 24 months, including 45 items; 2 to 4 years, including 21 items; and versions for 5 to 7, 8 to 12, and 13 to 18 years, including 23 items each. The PedsQL has been widely used across the globe in children aged 0 to 18 years.^1,8,9^ The questionnaires for children aged 2 to 18 years have been translated and validated in the South African population^10^ and have been used in many clinical studies, including those of cancer,^11^ juvenile idiopathic arthritis, and multisystem inflammatory syndrome in children,^12,13^ HIV and tuberculosis meningitis,^14^ epilepsy,^15^ and across multiple health groups.^16,17^ The Infant Scales (1-12 and 13-24 months)^18^ are, however, less frequently used and, to our knowledge, have not been validated in South Africa.

There has been recent interest from two separate groups of researchers in developing a health-state classification system from the PedsQL to allow for future valuation to develop a utility score.^9,19^ The classification systems derived by DeLuca et al.^9^and Kwon et al^19^ have been derived from the Longitudinal Study of Australian Children and are suggested for application to the versions for children aged 2 to 18 years. The classification system suggested by DeLuca includes 7 items, and Kwon’s suggests 4 items with an overlap of 3 items: “pain,” “other kids not wanting to be friends,” and “keeping up with schoolwork.” Further work is warranted to test these descriptive systems across disease groups and settings. This does, however, indicate the increasing interest in preference-weighted measures across the pediatric age range.

The EuroQol Research Foundation has ongoing research to develop the EuroQol Toddler and Infants (EQ-TIPS) measure for children age 0 to 47 months.^20^ This measure is considered experimental and implies that the development thereof is ongoing.^20^ Version 2 of the EQ-TIPS-3L includes 6 dimensions: movement, play, pain, communication, social interaction, and eating. Version 2 has shown good psychometric results in South Africa,^21–25^ China,^26,27^ and Australia.^28^ Although no utility values are available for the EQ-TIPS yet, each EQ-TIPS health state will likely be associated with an EQ value, which will allow for the calculation of quality-adjusted life-years for use in economic evaluations of healthcare interventions for this age group.

The PedsQL and EuroQol measures (EQ-TIPS and EQ-5D-Y) are currently the only two outcome measures, potentially amenable to preference-weighting, which will allow for measurement of HRQoL across the pediatric age range of 0 to 18 years. Thus, this study aims to compare the performance of these measures in a sample of South African infants and toddlers with a range of chronic health conditions.

## METHODS

### Study Design and Setting

EQ-TIPS-3L and PedsQL data collected between February 2022 and September 2025, from caregivers of children between the ages of 0 and 4 years with chronic health conditions, were pooled for analysis. Participants consisted of caregivers, typically a parent, of infants and toddlers attending a tertiary pediatric hospital in the Western Cape, South Africa. Red Cross War Memorial Children’s Hospital treats over 250 000 patients a year in both the acute and chronic services. Children with the following diagnoses were included: oncological or hematological disease, epilepsy, neuromuscular disease, cystic fibrosis, dependence on technology for breathing (tracheostomy and/or ventilation), gastrointestinal tract disease (including liver disease and/or transplantation), and renal disease (including dialysis and transplantation).

Psychometric studies lack standardized guidelines for calculating sample size.^29^ The minimum sample sizes were thus informed by the 2019 COSMIN study design checklist, which suggests that at least 100 patients in each subgroup should provide very good data for convergent validity.^30^ As this data informed a larger study across the age range of 0 to 18 years, larger samples were targeted by condition group across the pediatric age range.

### Instruments

**EuroQol Toddler and Infant Population-3 Level version 2.0. (EQ-TIPS-3L):** The EQ-TIPS is an experimental measure developed by the EuroQol Group to measure and value HRQoL in infants and toddlers. EuroQol experimental measures are tools which are still under development and may change as development proceeds.^20^ The EQ-TIPS is a proxy-report instrument completed by the primary caregiver, typically the parent.

The EQ-TIPS-3L version 2.0 descriptive system currently comprises 6 dimensions: movement, play, pain, communication, social interaction, and eating. Each dimension of the EQ-TIPS-3L has 3 levels of severity corresponding to “no problems,” “some problems,” and “a lot of problems.” The response on each dimension is defined as a 1-digit number that expresses the level selected for that dimension, where “no problems” is assigned a “1” and the most extreme level a “3.” The digits for each of the 6 dimensions can be combined into a 6-digit number that describes the younger patient’s health state. For example, the health state 111233 would represent “no problems” on the dimensions of movement, play, and pain; “a little bit of a problem” with social interaction; and “a lot of problems” with communication and eating.

The EQ-TIPS-3L does not yet have a preference-based score; therefore, a level sum score (LSS) like that used for the EQ-5D^31^ was used to describe the responses on the descriptive system where the level labels are treated as numeric data with the EQ-TIPS-3L score ranging between 6 and 18. Considering that there are only 6 EQ-TIPS dimensions, a LSS was not calculated for participants with any number of missing responses. A higher number indicates a worse HRQoL. The EQ-TIPS-3L was designed to be amenable to developing preference weights in the future.

The Visual Analogue Scale (VAS) is used to record the proxy’s view of how good or bad the infant’s health is overall on the day of questionnaire completion. The EQ VAS is vertical with endpoints labeled “the best health you can imagine” and “the worst health you can imagine.” A higher EQ VAS score indicates better health.

While the EQ-5D-Y-3L and EQ-5D-Y-5L are recommended for proxy completion between 4 to 18 years,^32^ the EQ-TIPS-3L dimensions are considered more appropriate and have been shown to perform better than the EQ-5D-Y-3L in 4-year-old South African children. Thus, the EQ-TIPS-3L was used for children aged 0 to 4 years in this study.

### PedsQL

**Pediatric Quality of Life Inventory 4.0 (PedsQL):** The age-appropriate PedsQL 4.0 generic measures for children aged 0 to 4 years were used for the proxy report. Each version has a variable number of items with the questionnaire, including 36 items for 1 to 12 months, 45 items for 13 to 24 months, and 21 items for 2 to 4 years. Responses for 1 to 12 months and 13 to 24 months were analyzed together, as the 36 items in the 1- to 12-month version are included in the 13- to 24-month version and are the same. The additional 9 items in the 13- to 24-month version were presented and marked as unique to the 13- to 24-month group. Each item is scored on a Likert scale of 0-4 (never a problem, almost never, sometimes, often, or almost always a problem). Items are reverse-scored and transformed to a 0-100 scale: 0 = 100, 1 = 75, 2 = 50, 3 = 25, 4 = 0. Dimension scores are calculated by a sum of the item scores divided by the total number of items. A total score is similarly generated by summing the dimension scores over the total number of dimensions, giving an overall HRQoL score. Scores for scales with more than 50% missing data were excluded from analyses. A higher PedsQL score indicates a better HRQoL.^33–35^

Kwon et al propose a 4-item scale for children aged 2 to 18 years to derive a future utility score. These items include “get aches and pains,” “feel sad/blue,” “other kids not friends,” and “keeping up with schoolwork.” The scores of these 4 items were averaged to generate a Kwon PedsQL GCS total score for further analysis. Of these 4 items, only pain or “having hurts or aches” was included in the Infant Scales.

DeLuca et al suggest 7 items to generate a classification system (PedsUtil) that could be used in the future to generate a utility score for children aged 2 to 18 years. Items from physical functioning include “participating in sport activity,” “pain,” and “fatigue.” Items from emotional functioning include “worrying about what will happen”; social functioning, “other kids not friends”; and school functioning, “keeping up with schoolwork” and “school absence.” Average scores were calculated for the DeLuca PedsUtil subscores and a total score. Of these 7 items, only pain (“having hurts or aches”) and fatigue (“feeling tired”) were included in the infant scales.

**Supplementary Table S1** compares the EQ-TIPS-3L and PedsQL properties (response scales, recall period, number of responses, and scoring).

### Study Procedure

Ethical approval was obtained from the University Human Research and Ethics Committee (HREC 740_2021). Approval was gained from all the relevant authorities. Caregivers of children aged 0 to 18 years receiving outpatient health care were recruited from the waiting rooms of specialist clinics at the children’s hospital. To avoid bias, all caregivers and children were given the opportunity to participate. Each caregiver agreed and consented to participate in the study. Responses from caregivers of children dependent on technology for breathing were collected on paper or electronic surveys and available in English, Afrikaans or isiXhosa.^25^ Data from all other caregivers was collected on handheld electronic devices, in English, and managed using REDCap electronic data capture tools hosted at the University of Cape Town.^36,37^ The researcher assisted with electronic competition, if needed, and/or was available for any clarification. The survey for children aged 0 to 4 years included information regarding the study and informed consent, EQ-TIPS-3L, PedsQL generic module, demographic and medical information for the child and caregiver, HRQoL and demographic information. Data from children 4 to 18 years will be presented elsewhere, as the EQ-TIPS is not appropriate for this age range.

### Statistical Analysis

**General performance and feasibility**: The EQ-TIPS-3L and PedsQL responses and descriptive data were summarized in terms of frequency of responses. For simplicity and standardisation of comparing the frequency of reporting problems between instruments with a different number of the responses these were collapsed into two categories “no/never a problem” and “any problem” (any other response) and were compared between the EQ-TIPS-3L and PedsQL. The feasibility was assessed by comparing the number of missing values across measures.

**Concurrent validity**: The concurrent validity of the EQ-TIPS-3L individual dimension scores was compared with the individual PedsQL items using Spearman correlations (r_s_). EQ-TIPS-3L LSS, EQ VAS, and PedsQL subscores and total scores (PedsQL, Kwon PedsQL GCS and DeLuca PedsUtil) were similarly compared using Spearman correlations (r_s_). Items and dimensions that were hypothesized to have moderate to strong correlations are presented in **Supplementary Table S2**. Hypotheses were developed by the study team, based on clinical judgement and conceptual overlap. Correlation coefficients were interpreted according to Cohen: 0.1-0.29 low association, 0.3-0.49 moderate association, and ≥0.5 high association.^38^

**Known group validity**: Mann-Whitney *U* tests were computed to ensure that there were no age-related differences by age group (0-24 months or 2-4 years) for the EQ-TIPS-3L LSS and total PedsQL scores. The health conditions included were heterogeneous, making severity comparison challenging. Thus, severity groups were categorized by EQ VAS score ≥80,^39^ and HRQoL scores were compared across the two groups with the Mann-Whitney *U* test.

All data analyses were conducted using SPSS Windows 30.0 (IBM SPSS Inc.), and significance was set at *P*≤.05.

## RESULTS

No caregivers of children dependent on technology for breathing completed the surveys in Afrikaans or isiXhosa. There were no exclusions from the other surveys due to English literacy. Data from 263 participants was available; however, data from 3 participants were excluded as over 50% of PedsQL responses were missing. No EQ-TIPS-3L data were missing from these 3 participants. These results thus report on data from a total of 260 children aged 0 to 24 months (n =111, 43%) and 2 to 4 years (n = 149, 57%). **Table 1** details the sociodemographic characteristics of the children and their caregivers. To note, neuromuscular disease and epilepsy were less frequently reported in the 0- to 24-month group, while gastrointestinal issues were more frequently reported compared with the 2- to 4-year group (χ^2^ = 18.04, *P* = .021). Most caregivers across both age groups were mothers; however, more of the caregivers in the 0- to 12-month group had completed tertiary level education (χ^2^ = 17.7, *P* = .002). As anticipated, caregivers spent significantly more time (≥10 hours) with younger children (0 to 24 months) (χ^2^ = 18.12, *P* = .001).

**Table 1.** Sociodemographic Characteristics of Children and Their Caregivers

	**0-24 mo, n (%) (N=111)**	**2-4 y, n (%) (N=149)**	χ**^2^**	***P* Value**
Child’s health condition				
Neuromuscular	2 (2)	11 (7)	18.04	**.021**
Epilepsy	4 (4)	11 (7)		
Cystic fibrosis	7 (6)	11 (7)		
Hematology	9 (8)	10 (7)		
Gastrointestinal tract disease	19 (17)	9 (6)		
Renal	20 (18)	28 (19)		
Oncology	18 (16)	35 (23)		
Technology for breathing	32 (29)	34 (23)		
Child’s gender				
Female	51 (46)	61 (41)	0.35	.420
Male	60 (54)	88 (59)		
Caregiver’s relationship to child				
Mother	90 (81)	121 (81)	4.84	.304
Father	11 (10)	17 (11)		
Grandmother	2 (2)	5 (3)		
Other	8 (7)	6 (4)		
Caregivers’ relationship status				
Married	51 (46)	66 (44)	6.65	.248
Single	38 (34)	54 (36)		
Life partner	15 (14)	23 (15)		
Divorced	0	3 (2)		
Widowed	2 (2)	2 (1)		
Prefer not to answer	4 (4)	1 (1)		
Caregivers’ highest level of education completed				
No schooling	0	3 (2)	17.17	**.002**
Primary school	28 (25)	29 (19)		
High school	41 (37)	86 (58)		
Tertiary	37 (33)	30 (20)		
Prefer not to answer	5 (5)	1 (1)		
Caregiver’s current employment				
Full-time employed	46 (41)	53 (36)	9.92	.128
Part-time employed	12 (11)	21 (14)		
Unemployed (not looking for work)	24 (22)	25 (17)		
Unemployed (looking for work)	19 (17)	43 (29)		
Student	4 (4)	4 (3)		
Retired	1 (1)	2 (1)		
Prefer not to answer	5 (5)	1 (1)		
Total household income from all sources per month				
<R5000	74 (42)	75 (50)	12.46	.086
R5000-R10 000	19 (17)	29 (19)		
R10 000-R15 000	13 (12)	16 (11)		
R15 000-R20 000	12 (11)	6 (4)		
≥R20 000	15 (14)	19 (13)		
I prefer not to answer	5 (5)	4 (3)		
Living conditions				
Informal housing without electricity and/or water	13 (12)	13 (9)	6.34	.274
Informal housing; with electricity and water	21 (19)	26 (24)		
Formal housing	72 (65)	98 (66)		
I prefer not to answer	5 (5)	2 (1)		
No. of children in caregiver’s care				
1	43 (39)	44 (30)	4.31	.230
2	32 (29)	53 (36)		
3	16 (14)	32 (21)		
4	8 (7)	14 (9)		
≥5	12 (11)	6 (4)		
No. of hours caregiver spent with child in past 24 hours				
0-3	2 (2)	9 (6)	18.12	**.001**
4-6	13 (12)	33 (22)		
7-10	15 (14)	34 (23)		
≥10	76 (68)	72 (48)		
Not answered	5 (5)	1 (1)		

Bolded text indicates significance, *P* ≤ .05.

**Figure 1.**
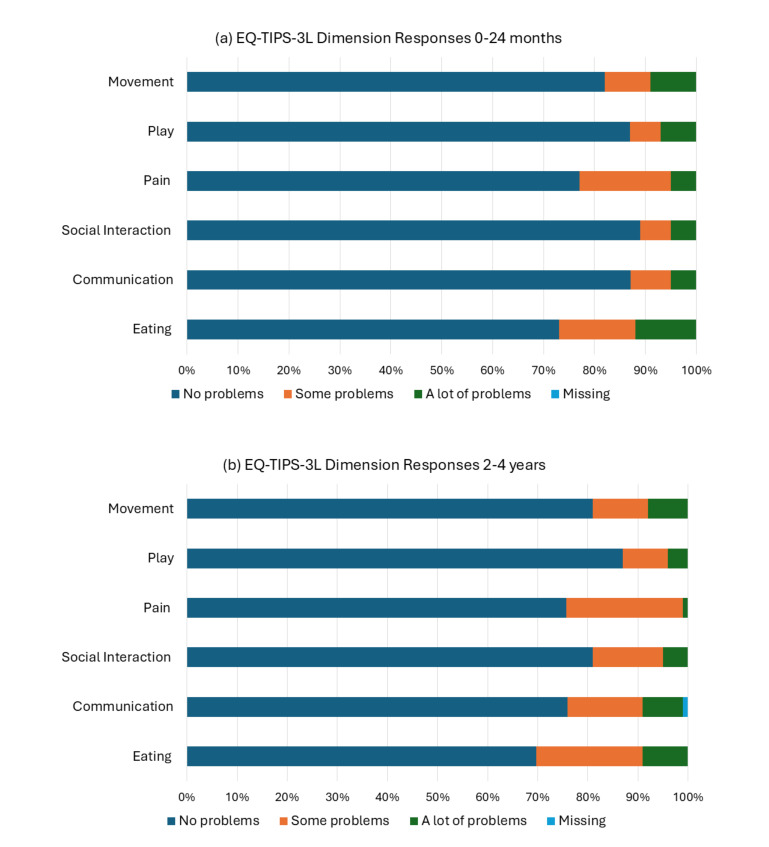
Frequency of EQ-TIPS-3L Dimension Responses for Children Aged 0 to 24 Months (**a**) and 2 to 4 Years (**b**)

**Figure 2.**
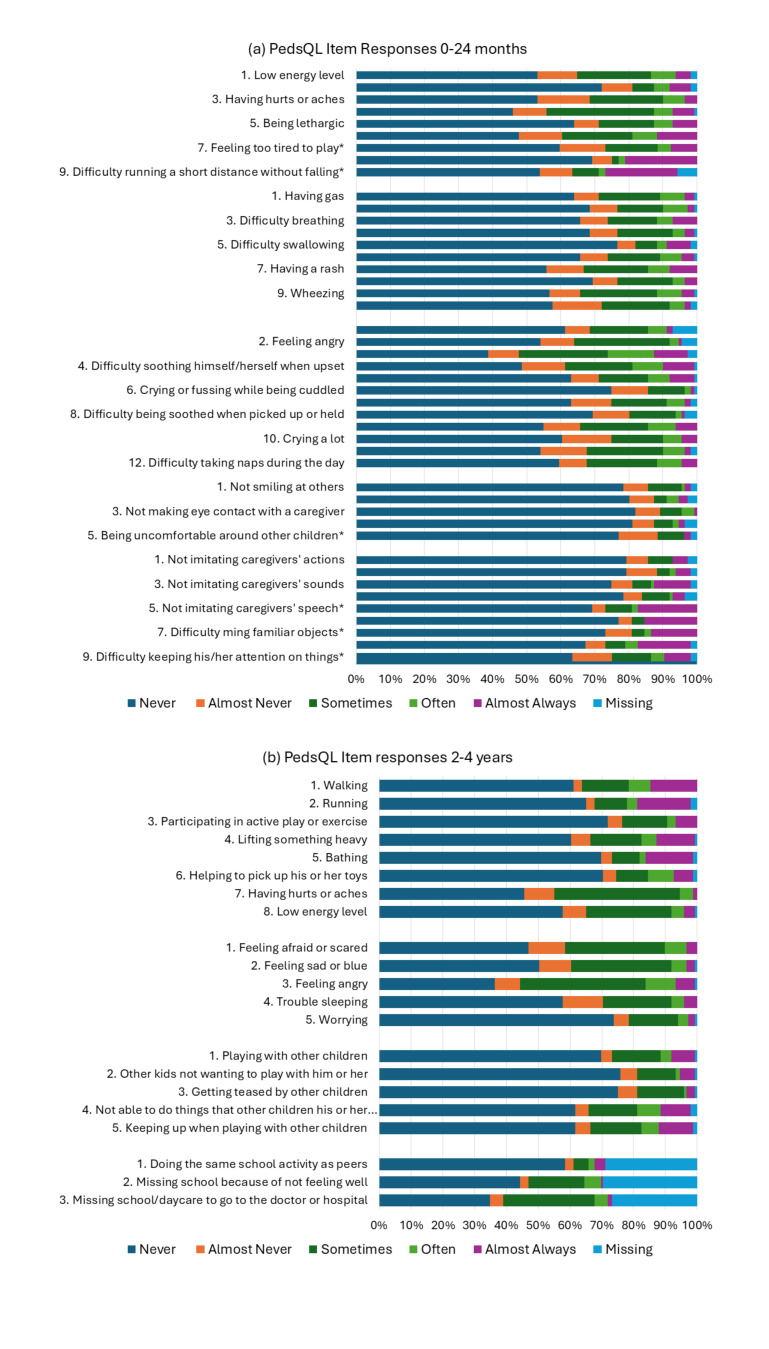
Frequency of PedsQL Item Responses for Children 0 to 24 Months (**a**) and 2 to 4 Years (**b**)

The EQ-TIPS-3L had the highest report of problems for the dimensions of eating and pain across the 0- to 24-month (**Figure 1a**) and 2- to 4-year groups (**Figure 1b**). There was an increase in problems reported with communication in the 2- to 4-year group compared with the 0- to 24-month group. There were missing responses for communication in the 2- to 4-year group only (1%).

The PedsQL similarly had high reporting of never having a problem across most items. For the 0- to 24-month group (**Figure 2a**), most problems (>50%) were reported in physical functioning for “feeling tired” and “resting a lot,” and in emotional functioning for “crying or fussing when left alone” and difficulty soothing himself/herself when upset.” “Crying or fussing when left alone” had the highest report of problem across all PedsQL items. “Feeling afraid or scared” (7%), difficulty running a short distance without falling” (13-24 months, 6%), and “feeling angry” (5%) had the highest report of missing items.

For the PedsQL (2- to 4-year version, **Figure 2b**), the highest reporting of problems was for “having hurts or aches,” feeling afraid or scared,” feeling sad or blue,” feeling angry,” and “missing school.” All 3 of the school functioning items had a high frequency of missing responses (range, 27%-30%). Most caregivers reported these items to the research assistant as the items were considered inappropriate because their children were not yet attending school. The high rate of problems reported may similarly be attributed to the child not yet attending school.

The authors hypothesized that the PedsQL and EQ-TIPS-3L would have 35 and 12 moderate to strong correlations for the 0- to 24-month and 2- to 4-year age groups, respectively (**Supplementary Table S2**). Only 6 of these items did not meet these criteria for the 0- to 24-month group, including EQ-TIPS-3L pain and PedsQL “crying a lot,” “being sick to his/her stomach,” and “difficulty breathing,” EQ-TIPS-3L communication and PedsQL “not imitating caregivers’ speech” and “difficulty pointing to his/her body parts when asked,” and EQ-TIPS-3L eating and PedsQL “having gas.” There were further moderate to strong correlations noted for the EQ-TIPS-3L LSS and all PedsQL subtotal and total scores, except the cognitive functioning subscore for the 0- to 24-month group (**Table 2**).

**Table 2.** EQ-TIPS-3L and PedsQL Spearman’s Correlations for Children Aged 0 to 24 Months and 2 to 4 Years

**PedsQL**	**EQ-TIPS-3L**						
**Movement**	**Play**	**Pain**	**Social Interaction**	**Communication**	**Eating**	**EQ-TIPS-3L LSS**	
**0-24 mo**							
PedsQL physical functioning subscore	**−0.439^b^**	**−0.467^b^**	**−0.413^b^**	−0.347^b^	−0.306^b^	−0.400^b^	−0.562^b^
1. Low energy level	−0.363^b^	−0.374^b^	−0.365^b^	−0.293^b^	−0.296^b^	−0.387^b^	−0.477^b^
2. Difficulty participating in active play	−0.476^b^	**−0.564^b^**	−0.397^b^	−0.484^b^	−0.418^b^	−0.355^b^	−0.542^b^
3. Having hurts or aches	−0.316^b^	−0.293^b^	**−0.535^b^**	−0.308^b^	−0.117	−0.319^b^	−0.402^b^
4. Feeling tired	−0.313^b^	−0.385^b^	−0.315^b^	−0.251^b^	−0.257^b^	−0.294^b^	−0.378^b^
5. Being lethargic	−0.314^b^	−0.390^b^	−0.323^b^	−0.349^b^	−0.272^b^	−0.233^a^	−0.356^b^
6. Resting a lot	−0.335^b^	−0.367^b^	**−0.305^b^**	−0.179	−0.238^a^	−0.315^b^	−0.440^b^
7. Feeling too tired to play^c^	−0.392^b^	**−0.425^b^**	−0.171	−0.312^a^	−0.388^b^	−0.196	−0.429^b^
8. Difficulty walking^c^	**−0.515^b^**	−0.535^b^	0.01	−0.341^a^	−0.390^b^	−0.376^b^	−0.482^b^
9. Difficulty running a short distance without falling^c^	**−0.495^b^**	−0.473^b^	−0.043	−0.258	−0.389^b^	−0.348^a^	−0.456^b^
PedsQL physical symptom subscore	−0.213^a^	−0.204^a^	**−0.330^b^**	−0.207^a^	−0.262^b^	**−0.436^b^**	−0.469^b^
1. Having gas	0.023	0.009	−0.065	0.073	−0.244^a^	**−0.098**	−0.072
2. Spitting up after eating	−0.134	−0.171	−0.175	−0.078	−0.111	**−0.617^b^**	−0.392^b^
3. Difficulty breathing	−0.193^a^	−0.175	**−0.188^a^**	−0.065	−0.229^a^	−0.270^b^	−0.283^b^
4. Being sick to his/her stomach	−0.121	−0.082	**−0.220^a^**	−0.107	−0.138	−0.212^a^	−0.312^b^
5. Difficulty swallowing	−0.335^b^	−0.317^b^	−0.199^a^	−0.223^a^	−0.274^b^	**−0.431^b^**	−0.472^b^
6. Being constipated	−0.091	−0.142	−0.234^a^	−0.280^b^	−0.251^b^	−0.229^a^	−0.215^a^
7. Having a rash	−0.034	0.006	−0.267^b^	−0.081	−0.051	−0.192^a^	−0.178
8. Having diarrhea	−0.037	−0.021	−0.11	−0.137	−0.166	0.017	−0.06
9. Wheezing	−0.11	−0.08	−0.223^a^	−0.166	−0.149	−0.217^a^	−0.293^b^
10. Vomiting	−0.248^b^	−0.281^b^	−0.230^a^	−0.187	−0.205^a^	**−0.446^b^**	−0.449^b^
PedsQL emotional functioning subscore	−0.175	−0.219^a^	−0.362^b^	−0.305^b^	−0.134	−0.366^b^	−0.381^b^
1. Feeling afraid or scared	−0.12	−0.096	−0.266^b^	−0.216^a^	−0.1	−0.240^a^	−0.248^a^
2. Feeling angry	−0.075	−0.005	−0.126	−0.19	−0.154	−0.187	−0.216^a^
3. Crying or fussing when left alone	−0.146	−0.141	−0.324^b^	−0.216^a^	−0.178	−0.231^a^	−0.279^b^
4. Difficulty soothing himself/herself when upset	−0.091	−0.188^a^	−0.298^b^	−0.142	0	−0.259^b^	−0.248^b^
5. Difficulty falling asleep	−0.111	−0.163	−0.424^b^	−0.301^b^	−0.158	−0.342^b^	−0.326^b^
6. Crying or fussing while being cuddled	−0.081	−0.1	−0.158	−0.139	−0.101	−0.147	−0.191^a^
7. Feeling sad	−0.186	−0.152	−0.228^a^	−0.148	−0.131	−0.298^b^	−0.202^a^
8. Difficulty being soothed when picked up or held	−0.186	−0.221^a^	−0.385^b^	−0.257^b^	−0.074	−0.438^b^	−0.390^b^
9. Difficulty sleeping mostly through the night	−0.101	−0.213^a^	−0.261^b^	−0.244^b^	−0.135	−0.212^a^	−0.258^b^
10. Crying a lot	−0.119	−0.131	**−0.295^b^**	−0.156	−0.06	−0.341^b^	−0.363^b^
11. Feeling cranky	−0.261^b^	−0.317^b^	−0.390^b^	−0.326^b^	−0.191^a^	−0.252^b^	−0.413^b^
12. Difficulty taking naps during the day	−0.134	−0.220^a^	−0.165	−0.335^b^	0.019	−0.136	−0.181
PedsQL social functioning subscore	−0.247^b^	−0.348^b^	−0.313^b^	**−0.515^b^**	−0.482^b^	−0.349^b^	−0.430^b^
1. Not smiling at others	−0.243^a^	−0.334^b^	−0.204^a^	**−0.489^b^**	−0.515^b^	−0.213^a^	−0.309^b^
2. Not laughing when tickled	−0.293^b^	−0.374^b^	−0.308^b^	**−0.373^b^**	−0.504^b^	−0.360^b^	−0.427^b^
3. Not making eye contact with a caregiver	−0.304^b^	−0.387^b^	−0.304^b^	**−0.539^b^**	−0.481^b^	−0.367^b^	−0.436^b^
4. Not laughing when cuddled	−0.275^b^	−0.351^b^	−0.369^b^	**−0.501^b^**	−0.476^b^	−0.335^b^	−0.439^b^
5. Being uncomfortable around other children^c^	-0.118	-0.183	-0.172	**-0.310^a^**	-0.152	-0.353^a^	-0.296^a^
PedsQL cognitive functioning subscore	-0.250^b^	-0.295^b^	-0.039	-0.372^b^	**-0.359^b^**	-0.176	-0.232^a^
1. Not imitating caregivers’ actions	-0.288^b^	**-0.315^b^**	-0.190^a^	**-0.451^b^**	-0.339^b^	-0.233^a^	-0.312^b^
2. Not imitating caregivers’ facial expressions	-0.274^b^	**-0.301^b^**	-0.188	**-0.377^b^**	-0.326^b^	-0.224^a^	-0.270^b^
3. Not imitating caregivers’ sounds	-0.200^a^	-0.237^a^	-0.11	**-0.358^b^**	-0.244^a^	-0.190^a^	-0.209^a^
4. Not able to fix his/her attention on objects	-0.244^a^	-0.269^b^	-0.144	-0.339^b^	-0.483^b^	-0.232^a^	-0.318^b^
5. Not imitating caregivers’ speech^c^	-0.067	-0.075	0.176	-0.211	**-0.261**	-0.13	-0.112
6. Difficulty pointing to his/her body parts when asked^c^	-0.294^a^	-0.318^a^	0.187	-0.017	**-0.269**	-0.05	-0.08
7. Difficulty naming familiar objects^c^	-0.326^a^	-0.359^b^	0.141	-0.265	**-0.412^b^**	-0.144	-0.262
8. Difficulty repeating words^c^	-0.269	-0.315^a^	0.155	-0.214	**-0.357^a^**	-0.119	-0.193
9. Difficulty keeping his/her attention on things^c^	-0.461^b^	-0.425^b^	-0.006	-0.327^a^	-0.507^b^	-0.290^a^	-0.402^b^
PedsQL total	-0.349^b^	-0.402^b^	-0.392^b^	-0.401^b^	-0.362^b^	-0.426^b^	-0.550^b^
**2 to 4 years**							
PedsQL physical functioning subscore	**-0.444^b^**	**-0.407^b^**	-0.289^b^	-0.392^b^	-0.319^b^	-0.322^b^	-0.513^b^
1. Walking	**-0.510^b^**	-0.374^b^	-0.249^b^	-0.299^b^	-0.262^b^	-0.151	-0.399^b^
2. Running	**-0.469^b^**	-0.366^b^	-0.242^b^	-0.331^b^	-0.275^b^	-0.223^b^	-0.403^b^
3. Participating in active play or exercise	**-0.516^b^**	**-0.490^b^**	-0.281^b^	-0.440^b^	-0.263^b^	-0.293^b^	-0.459^b^
4. Lifting something heavy	-0.341^b^	-0.391^b^	-0.212^b^	-0.338^b^	-0.324^b^	-0.325^b^	-0.402^b^
5. Bathing	-0.406^b^	-0.417^b^	-0.134	-0.393^b^	-0.210^a^	-0.180^a^	-0.328^b^
6. Helping to pick up his or her toys	-0.417^b^	-0.344^b^	-0.118	-0.353^b^	-0.389^b^	-0.306^b^	-0.407^b^
7. Having hurts or aches	-0.156	-0.156	**-0.379^b^**	-0.209^a^	-0.031	-0.284^b^	-0.319^b^
8. Low energy level	-0.258^b^	-0.286^b^	-0.346^b^	-0.333^b^	-0.198^a^	-0.354^b^	-0.400^b^
DeLuca PedsUtil physical functioning	-0.370^b^	-0.370^b^	-0.402b	-0.383^b^	-0.185^a^	-0.398^b^	-0.498b
PedsQL emotional functioning subscore	-0.096	-0.148	-0.313^b^	-0.269^b^	-0.259^b^	-0.152	-0.326^b^
1. Feeling afraid or scared	-0.064	-0.12	-0.235^b^	-0.190^a^	-0.149	-0.191^a^	-0.244^b^
2. Feeling sad or blue	-0.038	-0.118	-0.184^a^	-0.147	-0.081	-0.09	-0.134
3. Feeling angry	-0.071	-0.08	-0.307^b^	-0.202^a^	-0.252^b^	-0.11	-0.274^b^
4. Trouble sleeping	-0.137	-0.203^a^	-0.205^a^	-0.243^b^	-0.242^b^	-0.151	-0.308^b^
5. Worrying	-0.255^b^	-0.214^b^	-0.202^a^	-0.341^b^	-0.276^b^	-0.087	-0.333^b^
DeLuca PedsUtil emotional functioning	-0.244^b^	-0.205^a^	-0.222b	-0.328^b^	-0.263^b^	-0.075	-0.331b
PedsQL social functioning subscore	-0.286^b^	**-0.335^b^**	-0.161^a^	**-0.470^b^**	-0.412^b^	-0.266^b^	-0.475^b^
1. Playing with other children	-0.314^b^	-0.371^b^	-0.097	**-0.506^b^**	-0.407^b^	-0.207^a^	-0.407^b^
2. Other kids not wanting to play with him or her	-0.177^a^	**-.254^b^**	-0.145	**-0.467^b^**	-0.298^b^	-0.138	-0.281^b^
3. Getting teased by other children	0.03	0.019	-0.197^a^	-0.188^a^	-0.101	-0.088	-0.195^a^
4. Not able to do things that other children his or her age can	-0.337^b^	-0.344^b^	-0.157	-0.444^b^	-0.447^b^	-0.308^b^	-0.525^b^
5. Keeping up when playing with other children	-0.385^b^	-0.404^b^	-0.194^a^	-0.447^b^	-0.377^b^	-0.325^b^	-0.502^b^
DeLuca PedsUtil social	-0.167^a^	-0.244^b^	-0.134	-0.452^b^	-0.285^b^	-0.126	-0.261b
PedsQL school functioning subscore	0.091	-0.023	0.047	-0.055	-0.178	-0.161	-0.153
1. Doing the same school activity as peers	-0.042	-0.104	0.009	-0.173	-0.279^b^	-0.228^a^	-0.300^b^
2. Missing school because of not feeling well	0.11	−0.094	0.03	−0.125	−0.265^b^	−0.125	−0.168
3. Missing school/daycare to go to the doctor or hospital	0.117	0.017	0.019	0.015	−0.087	−0.121	−0.077
DeLuca PedsUtil school functioning	0.087	−0.088	0.016	−0.14	−0.279^b^	−0.181	−0.229a
PedsQL total score	−0.350^b^	−0.380^b^	−0.283^b^	−0.481^b^	−0.446^b^	−0.340^b^	−0.565^b^
Kwon PedsQL GCS total score	−0.146	−0.221^b^	−0.295^b^	−0.371^b^	−0.232^b^	−0.294^b^	−0.377^b^
DeLuca PedsUtil total	−0.306^b^	−0.342^b^	−0.331^b^	−0.474^b^	−0.328^b^	−0.362^b^	−0.519^b^

Values in bold were hypothesized to have a medium to strong correlation.

^a^*P* < .05.

^b^*P* < .001.

^c^Questions included in the 13- to 24-month PedsQL version only.

One of the hypothesized correlations was not met for the 2- to 4-year group: EQ-TIPS-3L play and PedsQL “other kids not wanting to play with him/her.” The EQ-TIPS-3L LSS showed moderate to strong correlations with all PedsQL subscore and total scores, except school functioning. EQ-TIPS-3L showed moderate to strong correlations with the DeLuca PedsUtil physical and emotional functioning subscores and total score only. The Kwon PedsQL GCS total score showed a moderate correlation with the EQ-TIPS-3L LSS (**Table 2**). The DeLuca PedsUtil scores showed strong correlations with the corresponding PedsQL subscores for physical functioning (r_s_ = 0.793), emotional functioning (r_s_ = 0.647), social functioning (r_s_ = 0.609), school functioning (r_s_ = 0.910) and total scores (r_s_ = 0.852). The Kwon total score showed a similarly high correlation with the PedsQL total score (r_s_ = 0.782). The Kwon PedsQL GCS and DeLuca PedsUtil total scores showed a strong correlation (r_s_ = 0.807).

Comparison of EQ-TIPS-3L and PedsQL scores by age group (**Table 3**) showed significantly higher HRQoL or lower reporting of problems for the emotional functioning subscore, social functioning subscore, and total PedsQL score for the 0- to 24-month group. No significant differences by age group were found for the EQ-TIPS-3L total scores or dimension scores.

**Table 3.** Comparison of EQ-TIPS-3L and PedsQL Scores by Age Group

	**0-24 mo (N = 111)**	**2-4 y (N = 149)**	**Mann-Whitney *U***	***P* Value**
VAS, median (IQR)	90 (70,100)	90 (70,100)	8376.5	.857
VAS ≥80, n (%)	76 (68)	101 (68)	χ^2^= 0.01	.907
EQ-TIPS-3L LSS, median (IQR)	6 (6,8)	7 (6,8)	8881.0	.273
EQ-TIPS-3L ceiling (111111), n (%)	61 (55)	71 (48)	χ^2^=1.36	.244
EQ-TIPS-3L floor (333333), n (%)	1 (1)	0		
PedsQL physical functioning subscore, median (IQR)	81 (63,100)	84 (69,97)	8359.5	.880
PedsQL physical symptoms subscore, median (IQR)	85 (73,95)			
PedsQL emotional functioning subscore, median (IQR)	82 (71,94)	80 (65,90)	7651.5	.237
PedsQL social functioning subscore, median (IQR)	100 (90,100)	90 (75,100)	5575.5	**<.001**
PedsQL cognitive/school functioning subscore, median (IQR)	100 (78,100)	83 (67,100)	4867.0	**.013**
PedsQL total, median (IQR)	87 (74,93)	82 (68,92)	7046.0	**.050**
PedsQL ceiling (total score 100), n (%)	3 (3)	8 (5)	χ^2^=1.12	.291
PedsQL floor (total score 0), n (%)	1 (1)	0		
Kwon PedsQL GCS total score, median (IQR)		83 (67, 100)		
DeLuca PedsUtil total score, median (IQR)		86 (71,96)		

Bolded text indicates significance, *P* ≤ .05.

The EQ-TIPS-3L LSS (*U* = 4776.5, *P*<.001), PedsQL total score (*U* = 10 633.5, *P* < .001), DeLuca PedsUtil total score (*U* = 3427.0, *P* < .001), and Kwon PedsQL GCS total score (*U* = 3229.5, *P* < .001) were all able to significantly differentiate between those with and without an EQ VAS score ≥80.

## DISCUSSION

Comparison of outcome measures that utilize different recall periods (EQ-TIPS-3L today vs PedsQL over the past month) has well-documented limitations, including more frequent reporting of problems with a longer recall period.^40–42^ Thus, it is unsurprising that EQ-TIPS-3L had a higher reporting of “no problems” across dimensions and overall (111111) compared with the PedsQL. This trend in reporting has been similarly reported when comparing the PedsQL with the EQ-5D-Y-3L in older children.^16,39,43^ The 5 response options on the PedsQL may further have contributed to a lower reporting of problems, and expanding the number of response options may be a future consideration for the EQ-TIPS-3L.^44–46^ The longer recall of the PedsQL is helpful for conditions where large fluctuations in symptoms are anticipated, whereas the short recall of the EQ-TIPS is well suited to measuring responsiveness to change.^40^

Considering the differences between the EQ-TIPS-3L (recall today and severity scale) and PedsQL (recall over the last month and frequency scale), the association between dimensions and items was good and exceeded the suggested 75% by COSMIN (COnsensus-based Standards for the selection of health Measurement INstruments.^47^ The comparison of these two instruments did, however, highlight some important differences. Difficulty running a short distance without falling (included on the 13- to 24-month PedsQL version) was frequently reported as “almost always a problem”; considering that the US Centers for Disease Control and Prevention only listed running as a milestone at 24 months, this may not be entirely relevant for the entire age group.^48^ Similarly, difficulty walking had a high frequency of reports of almost always a problem for the 13- to 24-month group, where, during normal development, children typically start stepping independently between 12 and 18 months.^48^ Thus, care should be taken in clinical settings to not overinterpret problems reported with these gross motor tasks in cohorts where problems are not anticipated. The high frequency of problems reported with eating on the EQ-TIPS-3L does not seem to be adequately reflected on the PedsQL for children aged 2 to 4 years, indicating that the version for younger children (13-24 months), which includes physical symptoms, may better capture this nuance. Alternatively, for children with anticipated eating difficulties, the EQ-TIPS-3L should be recommended. Conversely, when considering the low correlation between PedsQL cognitive/school functioning subscore and the EQ-TIPS-3L LSS, there is an indication that the EQ-TIPS-3L may not adequately assess cognitive functioning in its descriptive system. Questions regarding cognitive functioning (13-24 months) may also be more appropriate for the 2- to 4-year group than school functioning. Cognitive development is a cornerstone of development in this age group and both instruments warrant further qualitative and quantitative testing in children with clinically proven impairments to ascertain whether they adequately assess problems with cognition.

The DeLuca physical functioning subscore seems to perform well with similar high correlations to EQ-TIPS-3L, Kwon PedsQL GCS total score, and PedsQL physical functioning score. This may, however, be attributed to the fact that the score is calculated on a high number of items (3/8 physical functioning items). The reliance of both the DeLuca and Kwon total scores on missing school may be a concern in this age group, with a high rate of missing responses, as many were likely not attending school yet. This would need to be tested in different regions and languages, but without clearer guidance, the report on these items may be ambiguous, as school is not yet compulsory (in most countries) at this young age. The inclusion of items with a high rate of missing responses raises concerns over the PedsQL feasibility and relevance in the clinical assessment for the 2- to 4-year age group. Cognitive interviews with caregivers are encouraged to explore why school functioning items for 2- to 4-year-olds had high rates of missing data and whether alternative items might improve relevance. The correlation between the DeLuca emotional, social, and school subscores with the corresponding PedsQL subscores was lower than anticipated. Although the Kwon and DeLuca total scores showed a strong association with the PedsQL total score and EQ-TIPS-3L LSS, further work is suggested to test the proposed health status classification system in diverse cultural settings and health conditions before it is adopted for preference-weighted valuation.

The analysis of performance across the age groups was reassuring for both the EQ-TIPS-3L and the PedsQL total scores, as previous work had indicated that there may be age-related reporting of problems on the EQ-TIPS-3L.^49^ Further work is encouraged on both instruments to test the psychometric results, including test-retest reliability and responsiveness, across diverse language groups, narrow age bands, and within health conditions with clinically defined severity criteria, as there was a signal from the PedsQL that problems with emotional, social, and cognitive functioning were less prevalent in younger children. Limiting inclusion to caregivers and children who were able to complete English surveys limits the generalizability of the results to the South African setting. Translation and cross-cultural adaptation of outcome measures should be prioritized within this study for future research.

## CONCLUSION

The measures had a similar distribution of responses despite EQ-TIPS-3L reporting for “today” on a severity scale and PedsQL for the “past one month” on a frequency scale. EQ-TIPS-3L eating and pain had a high report of problems, and the former had a strong association with PedsQL physical symptoms only. Furthermore, the 2- to 4-year version had a high report of missing items for school functioning; thus, the 13- to 24-month PedsQL scale may be more appropriate for children aged 2 to 4 years who are not yet attending school and have problems with eating. As the association between PedsQL cognitive functioning and the EQ-TIPS-3L was low, further research is needed on whether this construct is considered missing on the EQ-TIPS V2.0 and whether this should be considered in future development of the instrument.

### Disclosures

J.V. is a member of the EuroQol Group.

### Ethics Approval and Consent to Participate

Ethical approval from the University Human Research and Ethics Committee (HREC 740_2021). All participants provided informed consent for participation and publication of the results.

### Consent for Publication

All participants provided informed consent for participation and publication of the results.

## Supplementary Material

Online Supplementary Material

## Data Availability

The dataset is available from the author on reasonable request.
