# Supplementary material for: Performance of the Experimental EuroQol Toddler and Infant Populations (EQ-TIPS) and PedsQL in Infants and Toddlers with a Health Condition
Source: J Health Econ Outcomes Res. 2025 Nov 17;12(2):209–20. doi: 10.36469/001c.145813 (PMC12629638; doi:10.36469/001c.145813)
Supplement: Online Supplementary Material [file jheor_2025_12_2_145813_310878.pdf]

Journal of Health Economics and Outcomes Research

## Online Supplementary Material

Performance of the Experimental EuroQol Toddler and Infant Populations (EQ-TIPS) and PedsQL in Infants and Toddlers With a Health Condition. *JHEOR*. 2025;12(2):209-220. doi:10.36469/jheor.2025.145813

**Table S1: Comparison of EQ-TIPS-3L and PedsQL Properties**

**Table S2: Hypothesized Medium to Strong Correlations Between EQ-TIPS-3L and PedsQL**

This supplementary material has been provided by the authors to give readers additional information about their work.

This is an open-access article distributed under the terms of the Creative Commons Attribution 4.0 International License (CCBY-4.0). View this license's legal deed at http://creativecommons.org/licenses/by/4.0 and legal code at http://creativecommons.org/licenses/by/4.0/legalcode for more information.

**Table S1.** Comparison of EQ-TIPS-3L V2.0 and PedsQL Properties

| Instrument | Age Range | No. of Items | Levels of Response | Response Scale | Recall Period | Scoring | | |
|---|---|---|---|---|---|---|---|---|
| | | | | | | **Preference Weights** | **Summary Score** | **General Health Score** |
| EQ-TIPS-3L | 0-4 y | 6 | 3 | Severity | Today | Development ongoing | Summed across all dimensions[a]; range, 6-18 | Visual Analogue Scale; range, 0-100 |
| PedsQL | 0-12 mo | 36 | 5 | Frequency | 1 mo | No | Reverse scored and summed with dimension subscores and total score; range, 0-100. | No |
| | 13-24 mo | 45 | 5 | Frequency | 1 mo | No | | |
| | 2-4 y | 21 | 5 | Frequency | 1 mo | Development ongoing | | |

[a]The summed score has several limitations: (1) the score assumes that the relative contribution of each dimension and each severity level is equal and (2) there are many health profiles with the same sum score. This is an interim solution to scoring until preference-weighted scores are developed.

**Table S2.** Hypothesized Medium to Strong Correlations Between EQ-TIPS-3L V2.0 and PedsQL

| EQ-TIPS-3L | PedsQL 0-12 Months and 12-24 Months | PedsQL 2-4 Years |
|---|---|---|
| Movement | 1. Physical functioning subscore<br>2. Difficulty walking[a]<br>3. Difficulty running a short distance without falling[a] | 1. Physical functioning subscore<br>2. Walking<br>3. Running<br>4. Participating in active play or exercise[b] |
| Play | 1. Physical functioning subscore<br>2. Difficulty participating in active play<br>3. Not imitating caregivers' actions[b]<br>4. Not imitating caregivers' facial expressions#<br>5. Feeling too tired to play[a] | 1. Physical functioning subscore<br>2. Social functioning subscore<br>3. Participating in active play or exercise[b]<br>4. Other kids not wanting to play with him or her[b] |
| Pain | 1. Physical symptom subscore<br>2. Having hurts or aches<br>3. Crying a lot<br>4. Being sick to his/her stomach<br>5. Resting a lot<br>6. Difficulty breathing | 1. Having hurts or aches |
| Social interaction | 1. Social functioning subscore<br>2. Not smiling at others<br>3. Not laughing when tickled<br>4. Not making eye contact with a caregiver<br>5. Not laughing when cuddled<br>6. Not imitating caregivers' actions<br>7. Not imitating caregivers' facial expressions<br>8. Not imitating caregivers' sounds[b]<br>9. Being uncomfortable around other children[a] | 1. Social functioning subscore<br>2. Playing with other children<br>3. Other kids not wanting to play with him or her |
| Communication | 1. Cognitive functioning subscore<br>2. Not smiling at others<br>3. Not making eye contact with caregiver<br>4. Not imitating caregiver's speech[a]<br>5. Difficulty pointing to his/her body parts when asked[a]<br>6. Difficulty naming familiar objects[a]<br>7. Difficulty repeating words[a] | |
| Eating | 1. Physical symptom subscore<br>2. Having gas<br>3. Spitting up after eating<br>4. Difficulty swallowing<br>5. Vomiting[b] | |

[a]These items are included in the 13- to 24-month PedsQL version only.
[b]These PedsQL items are hypothesized to correlate with multiple EQ-TIPS-3L dimensions.
